# Tiefe Hirnstimulation bei neurologischen und psychiatrischen Erkrankungen

**DOI:** 10.1007/s00115-021-01079-z

**Published:** 2021-02-25

**Authors:** Stephan Klebe, Volker Coenen

**Affiliations:** 1 Klinik für Neurologie, Universitätsmedizin Essen, Hufelandstr. 55, 45147 Essen, Deutschland; 2 Abteilung Stereotaktische und Funktionelle Neurochirurgie, Klinik für Neurochirurgie, Neurozentrum, Universitätsklinikum Freiburg, Breisacher Str. 64, 79106 Freiburg, Deutschland

**Keywords:** Idiopathisches Parkinson-Syndrom, Dystonie, Tremor, Psychiatrische Erkrankungen, Zielpunkt, Parkinsons Disease, Dystonia, Tremor, Psychiatric diseases, Target selection

## Abstract

Die invasive Hirnstimulation (tiefe Hirnstimulation [THS], „deep brain stimulation“ [DBS]) ist mittlerweile ein etabliertes Therapieverfahren bei einer Reihe neurologischer Erkrankungen insbesondere Bewegungsstörungen. Die Anzahl der mit einer THS versorgten Patienten steigt stetig, die technische Entwicklung der THS-Systeme schreitet voran und neue Indikationen werden aktuell in Studien überprüft. Im folgenden Beitrag soll ein Überblick über die aktuellen Indikationen und ein Ausblick auf zukünftige Entwicklungen der THS bei Bewegungsstörungen und psychiatrischen Erkrankungen gegeben werden.

## Abstract (en)

Invasive deep brain stimulation (DBS) has become an established treatment procedure for a number of neurological diseases, especially movement disorders. The number of patients treated with DBS is continuously increasing, the technical development of DBS systems is progressing and new indications are currently being tested in studies. This review gives an overview of the current indications and an outlook on future developments of DBS in movement disorders and psychiatric diseases.

## Historisches

Bereits in der Mitte des letzten Jahrhunderts wurden viele Patienten mit einem idiopathischen Parkinson-Syndrom (iPD) operativ behandelt. Die chirurgische Therapie bestand zumeist aus einseitigen Läsionen in Bereichen des Thalamus („Thalamotomie“) oder des Globus pallidus („Pallidotomie“). Das Zielsymptom dieser Operationen bestand in einer Besserung des krankheitsassoziierten Tremors, aber auch des Rigors bei iPD-Patienten. Leksell berichtete in den 1960iger-Jahren von einer Besserung durch Thermoläsionen im Pallidum von bis zu 80 % [1]. Einen Einschnitt für die operative Therapie des iPD stellte die Einführung der medikamentösen Therapie mit L‑Dopa in den 1960iger-Jahren dar. Dadurch wurden nur noch vereinzelt Patienten mit einem im Vordergrund stehenden Tremor mit einer einseitigen Thalamotomie im Nucleus ventralis intermedius (VIM) versorgt, bei denen ein schlechtes Ansprechen auf die L‑Dopa-Therapie vorlag. Ein wichtiger Punkt auf dem Weg zur THS war Mitte der 1980iger-Jahre die Beobachtung, dass die läsionelle Therapie im posteroventralen Teil des Globus pallidus internus (GPi) nicht nur die motorischen Symptome des iPD, sondern auch L‑Dopa-induzierte Dyskinesien bessert [2]. Etwa zur gleichen Zeit legten der Neurochirurg Alim Benabid und der Neurologe Pierre Pollak in Grenobel/Frankreich die Grundlage für die moderne THS. Es konnten vor allem durch ihre Arbeiten sowohl die Effektivität, Sicherheit und Überlegenheit der bilateralen VIM-THS bei Tremor [3] und später der positive Effekt der GPi- [4] und der Nucleus subthalamicus(STN)-THS [5] auf die motorischen Kardinalsymptome des iPD gezeigt werden. Seit der Einführung der THS in die moderne Therapie der Bewegungsstörungen wurden somit > 150.000 Patienten behandelt.

## Komponenten der THS

Die Komponenten des THS-Systems bestehen aus den Elektroden, dem Impulsgeber (Generator; IPS) und der Verbindung zwischen den beiden genannten. Der IPS selbst kann von außen über ein Programmiergerät angesteuert werden. Bei den IPS war eine wichtige Neuerung in den letzten Jahren die Einführung von wiederaufladbaren Geräten. Dadurch ist der IPS-Wechsel laut Herstellerangaben erst nach 15 bis 20 Jahren notwendig. Allerdings muss der Patient hier in der Lage sein, den IPS selbstständig zu laden. Der IPS selbst wiegt ca. 50–60 g. Die ursprünglichen Elektroden bestanden aus vier Ringkontakten, die über eine Länge von 7,5–10,5 mm angeordnet sind und eine radiale Ausbreitung des Stroms ermöglichen. Die wesentliche Neuerung bei den Elektroden war die Einführung segmentierter Kontakte, die eine zusätzliche Untergliederung der Kontakte mit sich bringt (direktionale Stimulation).

## Operatives Vorgehen

Der Erfolg der THS hängt bei allen Indikationen vor allem von der korrekten Auswahl der Patienten, der Identifikation der Zielsymptome und der optimalen Platzierung der Elektroden ab. Dieses Sweet-spot-Konzept und damit das postoperative Outcome wurde für mehrere Zielpunkte (z. B. STN, GPi) gezeigt [6, 7].

**Figure 1:**
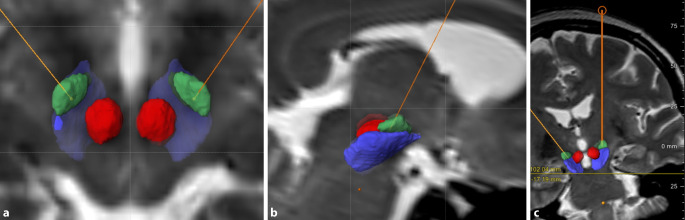

Die Operation erfolgt im Rahmen eines stereotaktischen Eingriffes, bei dem die Zielpunktkoordinaten und der Zugangsweg zuvor mithilfe einer kraniellen Magnetresonanztomographie (MRT) und einer speziellen Bildgebungssoftware millimetergenau berechnet werden (Abb. 1). Im Operationssaal sind in den meisten Zentren sowohl die operierenden Neurochirurgen als auch Neurologen anwesend; bei psychiatrischen Indikationen demzufolge die Psychiater. Dem Neurologen kommt im Operationssaal bei den Bewegungsstörungen die Aufgabe zu, den Patienten beim Erreichen des Zielpunktes zu untersuchen und die Wirkung der Neurostimulation bereits im Operationssaal zu überprüfen. In vielen Zentren werden zusätzlich – und bei bestimmten Zielpunkten – für den Patienten nicht spürbare elektrophysiologische Ableitungen durchgeführt, was die Genauigkeit der Operation erhöht. Im Anschluss wird die Elektrode über ein Kabel subkutan mit dem IPS verbunden, der wie ein Herzschrittmacher unter die Haut im Bereich des Brustmuskels oder abdominal eingesetzt wird. Ob die Operation in Vollnarkose, in reduzierter Narkosetiefe oder als Wachoperation durchgeführt wird, hängt von dem Zielpunkt, der zugrunde liegenden Erkrankung und der Präferenz des Patienten ab.

## Wirkweise der THS

Trotz Erfahrung mit der THS seit > 30 Jahren sind die genauen Mechanismen und die genaue Funktionsweise der THS nach wie vor nicht vollständig verstanden. Verschiedene Hypothesen wurden postuliert. Der hauptsächliche Effekt der THS besteht in einer Modulation pathologischer Aktivität zentraler neuronaler Netzwerke. Dabei erzeugen die elektrischen Felder der THS eine frequenzabhängige funktionelle Inhibition. Den myelinisierten Axonen kommt dabei nach herrschender Lehrmeinung die größte Rolle zu, da sie im Gegensatz zu Zellkörpern und dünnen unmyelinisierten Axonen eher auf elektrische Stimuli mit Aktivierung reagieren [8]. Die afferenten und efferenten axonalen Effekte der THS können somit intrinsische Signale funktioneller neuronaler Netzwerke eliminieren oder maskieren [9]. Welche neuronalen Elemente reagieren, hängt allerdings von weiteren Randbedingungen wie der Lage zur Stimulationselektrode, der Ausrichtung des Axons, Membraneigenschaften und den Stimulationsparametern ab. Das Einsetzen der Wirkung kann akut nach einigen Sekunden bis Minuten nach Beginn der Stimulation (z. B. Bradykinese, Tremor) oder erst nach Wochen oder Monaten (z. B. dystone oder axiale Symptome) erfolgen [10]. Die akuten Effekte werden vermutlich durch direkte elektrophysiologische Mechanismen erzeugt, während die chronischen Wirkungen über synaptische Modifikationen vermittelt werden [11].

## Indikationen

Insgesamt wurde zwischen 1998 und 2010 für fünf neuropsychiatrische Erkrankungen eine Zulassung für die THS erteilt (iPD, primäre und sekundäre Dystonien, Tremor, Epilepsie und Zwangserkrankungen; Tab. 1). Die Indikationsstellung und der gewählte Zielpunkt sollten immer interdisziplinär zwischen den beteiligten Fachdisziplinen (Neurochirurg/Stereotaktiker, Neurologe, Psychiater, Neuropsychologe) erfolgen. Dabei steht im Vordergrund, solche Patienten zu identifizieren, bei denen der mögliche Erfolg einer THS höher als das mögliche Operationsrisiko einzuschätzen ist. Wichtig ist zudem, bei den einzelnen Indikationen mit den Patienten und den Angehörigen sowohl über die Chancen der THS, aber auch über die Grenzen zu sprechen.

**Table 1:** 

	Etablierte Therapie	Zielpunkt	Ausgewählte Publikationen
Idiopathisches Parkinson-Syndrom	Ja	Nucleus subthalamicus (STN)	[13, 15]
		Globus pallidus internus (GPi)	[16]
Tremor bei idiopathischem Parkinson-Syndrom	Ja	STN oder Nucleus ventralis intermedius (Vim)	[60]
Essenzieller Tremor	Ja	Vim	[61]
Dystonie	Ja	GPi	[25]
Epilepsie	Ja^a^	Nucleus anterior thalami (ANT)	[62]
Zwangserkrankung (OCD)	(Ja)/nein^b^	Ventrale Kapsel/ventrales Striatum (VC/VS)	[63]
Depression	Nein	z. B. cg25/SCG	[49]
Alzheimer-Demenz	Nein	z. B. Fornix	[64]

^a^Zugelassen für fokale und sekundär generalisierende Epilepsie

^b^CE für DBS besteht, jedoch klare Einstufung als bisher experimentell durch die DGPPN

## Idiopathisches Parkinson-Syndrom

Das iPD stellt die häufigste Indikation für eine THS dar. In den letzten Jahren hat sich gezeigt, dass bei iPD-Patienten eine THS in Erwägung gezogen werden sollte, sobald im Verlauf der Erkrankung motorische Komplikationen („Wirkfluktuationen“) oder motorische Nebenwirkungen der Behandlung (Überbewegungen/Dyskinesien) unter der dopaminergen Therapie auftreten. Zusätzlich scheint die THS auch eine Option bei iPD-Patienten mit Impulskontrollstörungen (z. B. exzessives Kaufverhalten, Spielsucht, erhöhte Libido) zu sein [12]. Es macht durchaus Sinn, mit den Patienten bereits früh im Krankheitsverlauf über die mögliche Therapieoption einer THS zu sprechen. Das spiegelt sich auch in der viel zitierten Early-Stim-Studie [13] wider, bei der die Patienten im Vergleich zu vorangegangenen Arbeiten [14, 15] eine kürzere Erkrankungsdauer (7,5 vs. 11–13 Jahre), ein niedrigeres Lebensalter (53 vs. 60 Jahre) und Wirkfluktuationen von maximal 3 Jahren hatten. Es gilt mit Ausnahme des Tremors die Regel, dass die THS nur auf solche Symptome ein gutes Ansprechen zeigt, die auf eine dopaminerge Therapie bei dem einzelnen Patienten ansprechen. Der in vielen Studien gezeigte positive Einfluss auf die Lebensqualität ist dabei ein Resultat aus der Kontinuität und Gleichmäßigkeit der Therapie [13, 15]. Neben den allgemeinen operativen Kontraindikationen kommen iPD-Patienten mit manifesten psychiatrischen Erkrankungen (z. B. schwere Depression, Demenz) nicht für eine THS in Betracht.

Für eine THS kommen beim iPD verschiedene Kerngebiete und/oder Faserstrukturen infrage. Am häufigsten wird aktuell der STN als Zielpunkt gewählt. Dabei konnte in einer rezenten Vergleichsstudie zwischen STN und GPi gezeigt werden, dass der STN eine bessere Reduktion der Off-Phasen bei einem vergleichbaren Risiko für neuropsychiatrische Komplikationen (Kognition, Stimmung, Verhalten) zeigt [16]. Eine Ausnahme stellen iPD-Patienten mit einem therapierefraktären Tremor dar, die im Einzelfall gut von einer THS im VIM und/oder im posterioren subthalamischen Areal (PSA) profitieren können. Durch eine Stimulation im VIM/PSA werden allerdings nicht die Bradykinese oder der Rigor beeinflusst.

**Figure 2:**
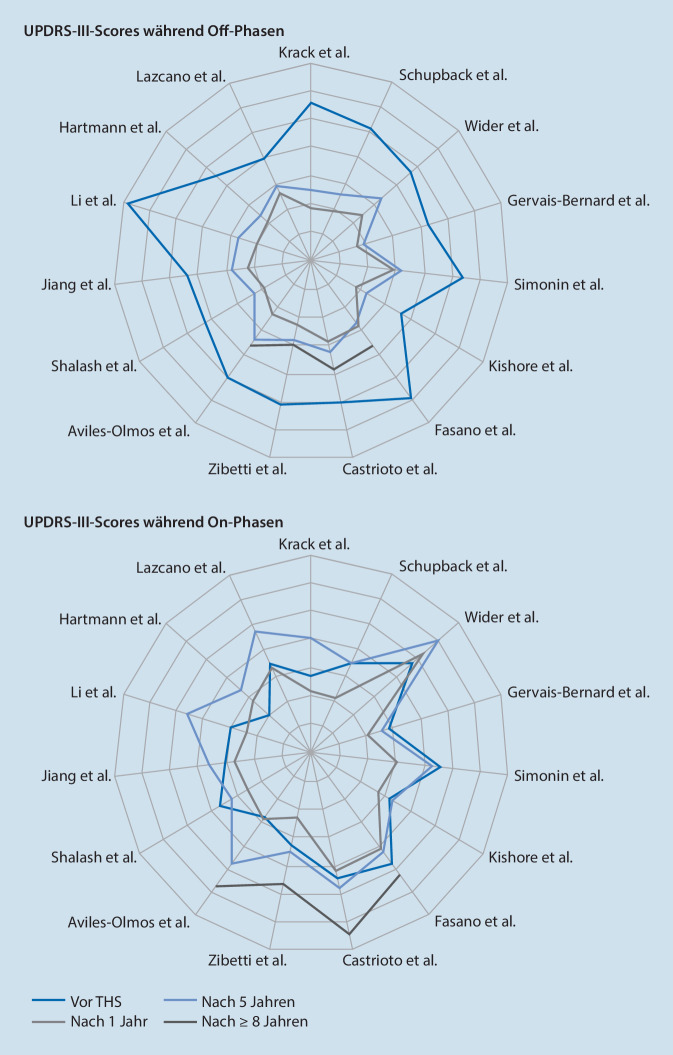

Der entscheidende Effekt der THS ist die verbesserte Lebensqualität der THS-Patienten im Vergleich zu medikamentös behandelten Patienten mit motorischen Komplikationen [13, 15, 17]. Daneben gelingt durch die THS bei iPD-Patienten eine Verbesserung der motorischen Symptome gemessen mit der Unified Parkinson’s Disease Rating Scale (MDS-UPDRS III) um ca. 50 %, eine Verkürzung der Off-Phasen um ca. 30–70 % und eine Reduktion der Dyskinesien um ca. 70 % [13–16, 18, 19]. Ein positiver Effekt der THS bei iPD-Patienten über längere Beobachtungszeiträume, teilweise seit > 10 Jahren, liegen mittlerweile ebenso vor ([17, 20]; Abb. 2).

## Dystonie

Nach dem iPD stellen die primären und sekundären Dystonieformen die zweithäufigste Indikation einer THS dar. Als Zielpunkt wird meistens der GPi gewählt [21, 22], wobei auch der STN positive Effekte auf eine Dystonie haben kann [23, 24]. Langzeitverläufe zeigen nach 5 Jahren ein weiterhin positives Ansprechen mit einer Verbesserung > 60 % auf der Burke-Fahn-Marsden Dystonia Rating Scale (BFMDRS) bei primären oder segmentalen Dystonien [25]. Positiv hervorzuheben ist dabei, dass die meisten primären Dystonien per se keinen neurodegenerativen Charakter haben und daher der natürliche Verlauf anders einzuordnen ist als bei iPD-Patienten. Allerdings müssen die Patienten darüber aufgeklärt werden, dass bereits fixierte Fehlhaltungen einer langen häufig über Monate andauernden postoperativen Einstellungsphase bedürfen. Die Patienten sollten idealerweise von einer langfristigen intensiven Rehabilitationsmaßnahme begleitet werden. Bei der Auswahl der primären Dystonien mit einer genetischen Ursache kommt der molekulargenetischen Diagnostik eine besondere Rolle zu. Bei einigen Formen (z. B. DYT-TOR1A [26], DYT-SGCE [27]) weiß man, dass diese sehr gut, andere (z. B. DYT-ATP13A1 [28]) gar nicht auf eine THS ansprechen.

Bei den fokalen Dystonien stellt die Behandlung mit Botulinumtoxin die Therapie der Wahl dar. Sollte diese keine ausreichende Wirkung haben, kann z. B. bei einer zervikalen Dystonie eine GPi-THS erwogen werden [22].

Die Ergebnisse der THS bei sekundären Dystonien sind sehr divergent. Sehr günstige Effekte werden für tardive Dystonien/Dyskinesien berichtet mit einer > 70 %igen Besserung der Symptome [29]. Bei neurodegenerativen Erkrankungen, die mit einer Dystonie assoziiert sind (z. B. „neurodegeneration with brain iron accumulation“, NBIA), bleibt die THS eine Einzelfallentscheidung. Bei Patienten mit infantiler Zerebralparese (CP) wird eine Verbesserung der Symptome um ca. 20 % beschrieben [30, 31]. Dabei ist zu berücksichtigen, dass es sich bei CP-Patienten um eine ätiologisch sehr heterogene Patientenpopulation handelt, worunter sich viele nichtaufgeklärte genetische Formen verbergen.

## Tremor

Ein Tremor kann bei unterschiedlichen neurologischen Erkrankungen und in unterschiedlicher Form auftreten (Ruhetremor, Aktionstremor [Halte‑, Intentionstremor]). Bei der THS stehen der Parkinson-Tremor (siehe Abschnitt iPD), der essenzielle Tremor (ET), aber auch seltene Indikationen (z. B. Tremor bei Multipler Sklerose [MS], Holmes-Tremor) im Vordergrund. Der ET stellt die häufigste neurologische Bewegungsstörung insbesondere bei Menschen im höheren Lebensalter dar. Allerdings ist er glücklicherweise meist nicht so stark ausgeprägt, dass er zu einer schweren Beeinträchtigung führt. Dadurch konsultieren nur ca. 30 % der Patienten mit einem ET einen Arzt [32]. Bei einer schweren Beeinträchtigung kann eine THS im VIM/PSA oder der kaudalen Zona incerta (cZI) eine aussichtsreiche Therapieoption sein. Dabei konnte eine Reduktion des Tremors der oberen Extremität bei einer THS im VIM/PSA von ca. 60 % und in kleineren Studien zur THS in der cZI um bis zu 90 % gezeigt werden [33, 34]. Für die cZI spricht auch die berichtete geringere Toleranzentwicklung über die Zeit, was einem Nachlassen der Wirkung auf den Tremor entspricht. Um dieser Toleranzentwicklung entgegenzuwirken, werden die Patienten angehalten, die Stimulation in der Nacht zu pausieren. Es fehlen aktuell allerdings Vergleichsstudien, die beide Zielpunkte (VIM/PSA und cZI) miteinander vergleichen. Die Datenlage zur Behandlung eines schweren Tremorsyndroms im Rahmen einer MS ist leider nicht sehr groß. Daher sollte die Entscheidung zu einer THS bei einem MS-assoziierten Tremorsyndrom nur bei ausgewählten Patienten getroffen werden. Mit in Betracht gezogen werden sollten (1) eine relative Stabilität der MS, (2) ein überwiegend isoliertes Tremorsyndrom und (3) eine Abgrenzung eines Intentionstremors und einer Ataxie, da diese nicht auf die THS ansprechen [35]. Bei der Chirurgie des Tremors kommen zunehmend bildgebende Verfahren wie die Darstellung des dentatorubrothalamischen Traktes (DRT) mittels der Diffusionstensorbildgebung zum Einsatz [36–39].

## Zwangserkrankung

Die THS bei Patienten mit einer therapierefraktären Zwangserkrankungen (OCD) wurde erstmalig 1999 veröffentlicht [40]. Der genaue Effekt der THS bei OCD ist unklar. Das kortikostriatothalamokortikale Netzwerk mit seinen Strukturen (orbitofrontaler Kortex, anteriores Zingulum, präfrontaler Kortex, ventrales Striatum) sind involviert und dienen als Zielstruktur für die THS [41]. Kontraindikationen sind signifikante psychiatrische Komorbiditäten (Psychose, akute manische Episode, Substanzmissbrauch, akutes Suizidrisiko, schwere Persönlichkeitsstörung). In einer kürzlich publizierten Metaanalyse von 31 Studien mit insgesamt 116 Patienten und 7 verschiedenen Zielpunkten wird über eine Besserung der Symptome von 45 % auf der Yale-Brown Obsessive-Compulsive Scale (Y-BOCS) berichtet [42]. Kritisch anzumerken ist allerdings, dass in den meisten Arbeiten nur über kleine Fallserien berichtet wurde, Kontrollgruppen fehlten und bislang keine Vergleichsstudien zu verschiedenen Zielpunkten existieren. Obschon das Verfahren mit einem THS-System der Firma Medtronic zugelassen ist, beruht diese Zulassung auf einem verkürzten Verfahren. Die Zulassung erfolgte auf Basis einer „humanitarian device exemption“, d. h., dass es keine wirkliche Zulassungsstudie gab [43]. Die DGPPN betrachtet die THS bei der Zwangserkrankung demzufolge auch weiterhin als „experimentell“.

## Postoperative Betreuung der Patienten

Die Nachbetreuung stellt ein wesentliches Qualitätsmerkmal in der Versorgung von THS-Patienten dar. Die Ersteinstellung des THS-Systems findet je nach Zentrum in den ersten 7 bis 14 Tagen nach der Operation oder nach dem Abklingen des Setzeffektes in der Regel stationär statt. Als Setzeffekt bezeichnet man, dass bei vielen Patienten eine Besserung der Symptome in den ersten Wochen nach der Operation vorliegt. Die genaue Ursache des Setzeffektes ist unklar. Denkbar ist, dass sich durch die mechanische Manipulation der THS-Operation allein ein positiver, allerdings nachlassender Effekt einstellt.

**Figure 3:**
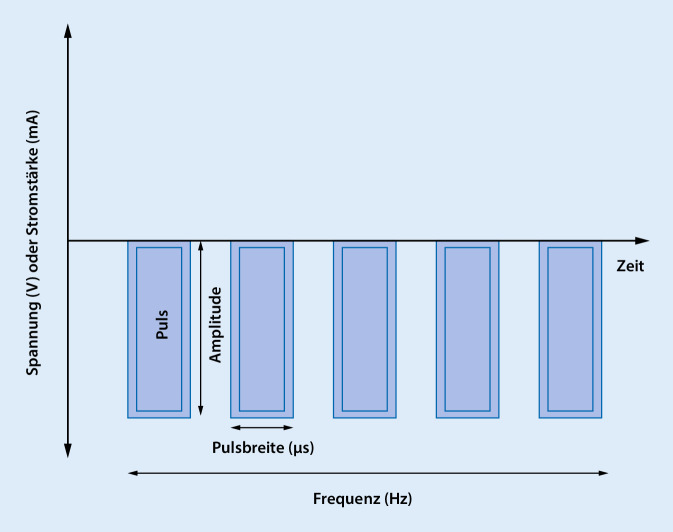

Bei der Ersteinstellung und Programmierung des IPS im Verlauf können die Amplitude des Impulses (Spannungsamplitude in Volt [V] oder Stromstärke in Milliampere [mA]), die Dauer der Einzelimpulse (in Mikrosekunden [μs]), die Frequenz der Einzelimpulse (in Hertz [Hz]) und die Polarität der einzelnen Kontakte modifiziert werden (Abb. 3). Stimulationsassoziierte Nebenwirkungen können die Folge einer falschen Stimulationseinstellung oder einer nichtoptimalen Elektrodenlage sein. Hierbei kann es z. B. zu Problemen mit dem Sprechen oder Gangstörung kommen, die meistens eine Anpassung der Stimulation und in den seltensten Fällen eine Kontrolle der Sondenlage erforderlich machen. Die Patienten sollten einen Ansprechpartner in den jeweiligen Zentren haben, da im Notfall jemand zur Verfügung stehen muss, der eine Stimulationseinstellung bzw. Änderung vornehmen kann. Bei der STN-Stimulation kommt es dabei typischerweise zu einer 50 %igen Reduktion des L‑Dopa-Äquivalents. Bei der GPi-Stimulation ist der Stimulationseffekt im Wesentlichen die Reduktion der durch Medikamente ausgelösten Dyskinesien. Demzufolge werden hierbei Medikamente in unveränderter oder leicht gesteigerter Dosierung weitergegeben.

**Figure 4:**
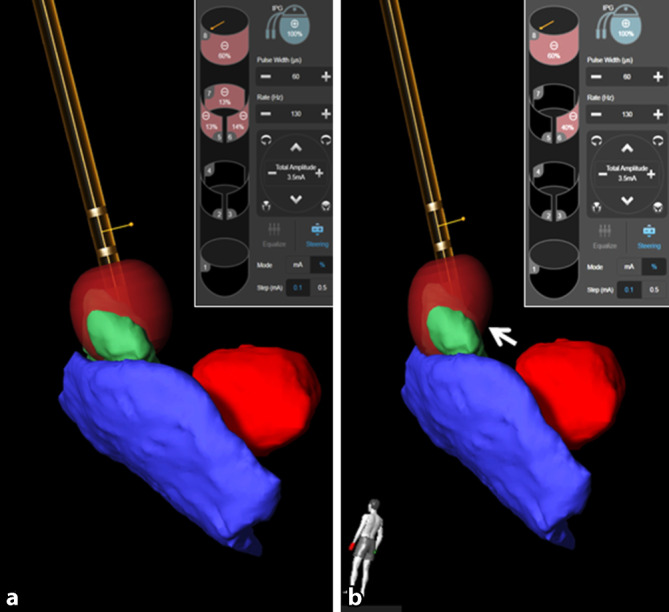

Durch den Einsatz neuer THS-Systeme und direktionaler Elektroden sind in den letzten Jahren neue Strategien bei der Programmierung der THS-Systeme verfolgt worden. In der Anfangszeit der THS hatte man sich bei der Einstellung und Anpassung insbesondere auf eine Änderung der Amplitude (in V oder mA) und der Polarität (monopolar vs. bipolare Einstellung) fokussiert. Dies basierte zumeist auf Expertenwissen und -erfahrung. Die modernen IPS ermöglichen, kürzere Impulsbreiten (μs) oder Änderungen der Frequenz (Hz) bei der Einstellung zu berücksichtigen. Durch die direktionalen Elektroden besteht zudem die Möglichkeit, Einfluss auf das aktivierte Gewebsvolumen („volume of tissue activated“, VTA) zu nehmen. Die Folge ist, dass neuroanatomische Strukturen und Fasertrakte besser in die Stimulation eingebunden oder herausgehalten werden können. Die Effektivität dieser Einstellungsstrategien auf die Symptome bzw. Nebenwirkungen bei Patienten mit iPD wurde in verschiedenen Studien bestätigt [44–46]. Einschränkend muss allerdings gesagt werden, dass die neuen Einstellungsoptionen bei komplexen Patienten in der Testung der Wirkung und Nebenwirkungen der THS sehr aufwendig und zeitintensiv sind. Hier werden zukünftig durch den Einsatz moderner bildgebender Verfahren und künstlicher Intelligenz Algorithmen entwickelt werden, die die Programmierung zumindest unterstützen (Abb. 4).

Neben der optimalen individuellen Stimulationseinstellung erfolgt in der postoperativen Phase die Anpassung der vorbestehenden Medikation. Gerade bei iPD-Patienten sollte eine zu schnelle Reduktion der dopaminergen Medikation aufgrund der Gefahr eines Dopaminentzuges mit z. B. Depression, Apathie nach der THS vermieden werden. Als Faustregel hat sich eine langsame Reduktion um 25 % zu Beginn und nach 3 bis 6 Monaten um ca. 50 % der L‑Dopa-Äquivalenzdosis bewährt. Im Durchschnitt können nach einer STN-Implantation im Durchschnitt bis zu 60 % der dopaminergen Medikation eingespart werden [47]. Dagegen bleibt nach einer Stimulation des Gpi die Medikation bei iPD-Patienten trotz einer guten Stimulationswirkung auf motorische Komplikationen unverändert [47]. Bei den Patienten mit einer Dystonie oder einem ET spielt eine Medikamentenanpassung keine große Rolle.

Über die gesamte Phase der Ersteinstellung (ca. 6 Monate nach der Operation) sind die geplanten ambulanten Kontakte mit den versorgenden Spezialambulanzen häufig. Nach der Einstellungsphase sollten weiterhin regelmäßige ambulante Kontakte mit den THS-Zentren gewährleistet sein. Ein Intervall von 3‑monatlichen Kontakten hat sich in vielen Zentren etabliert.

## Neue Indikationen

Im Rahmen der neuen Indikationen sind sicherlich die Behandlung therapierefraktärer Depression sowie der Alzheimer-Demenz zu nennen. Die THS bei der Depression wurde in neuerer Zeit als erstes von Helen Mayberg (Emory University, Atlanta, GA, USA) gemeinsam mit Andres Lozano (Toronto, Kanada) durchgeführt [48]. Neben der initialen Zielregion des subgenualen Zingulums wurde eine ganze Reihe weiterer Zielpunkt getestet. Tatsächlich sind zwei industriebetriebene Studien aufgrund des im Nachhinein erwartbar nicht zu erreichenden primären Endpunktes gestoppt worden [49, 50]: Inzwischen sind nämlich eine Reihe von Fehlern im Studiendesign dieser Studien erkannt worden und auch hier finden neue Bildgebungsverfahren Einzug [51]. Arbeitsgruppen arbeiten international an der Etablierung der Indikation der THS für die Depression [52–55]. Für die Alzheimer-Erkrankung sind mehrere Studien unter der Leitung der Kollegen aus Toronto publiziert worden [56, 57]. Die Ergebnisse sind schwierig zu interpretieren, wurden aber von der Industrie als so gut bewertet, dass inzwischen eine internationale multizentrische Studie zur Fornixstimulation in Durchführung ist.

## Technische Entwicklungen

Eine zukünftige Strategie der THS wird darauf abzielen, die präoperative Planung (siehe Abb. 1), die operativen Techniken und die postoperative Einstellung zu verbessern. Künstliche Intelligenz und Computersimulationen in der Bildgebung [6] werden dabei eine wichtige Rolle spielen. Zusätzlich werden neue Stimulationsformen wie die adaptive Stimulation zum Tragen kommen. Die bisherige THS basiert auf der chronischen Stimulation, die unabhängig der neuronalen Aktivität appliziert wird. Bei den adaptiven Verfahren wird die Stimulation nur noch bei Bedarf angewandt. Diese „Feedback“-kontrollierte Stimulation wird als „closed loop“ bezeichnet, da es sich um ein geschlossenes System handelt. Dabei kommt es zu einem Austausch zwischen Symptomen des Patienten (z. B. Akinese, Tremor), der dem Symptom zugrunde liegenden neuronalen Aktivität und eine darauf angepasste Reaktion des Stimulationssystems [58]. Der Trigger eines Closed-loop-Systems kann sowohl über die klinischen Symptome der Peripherie (z. B. Tremor gemessen über Sensoren an den Extremitäten) als auch über zentrale Signale (z. B. Betaaktivität in den Basalganglien) kommen [59]. Alle genannten Neuerungen werden zusätzlich das pathophysiologische Verständnis der THS verbessern.

## Ausblick

Die THS wird aufgrund der sich in Entwicklung befindlichen Techniken und erweiterten Indikationen auch zukünftig einen festen Platz in der Behandlung von Bewegungsstörungen und zunehmend auch neuropsychiatrischen Erkrankungen haben. Spannend zu beobachten wird sein, wie sich ablative und damit irreversible Verfahren wie die MRT-gesteuerte fokussierte Ultraschall(MRgFUS)-Technik entwickeln. Damit eröffnen sich möglicherweise neue therapeutische Optionen, die weniger invasiv als die THS sind. Hier sind vergleichende Studien (z. B. THS vs. MRgFUS) notwendig, um diese Techniken mit einander zu vergleichen. Vorstellbar ist, dass ausgewählte Patientengruppen mehr von der THS und andere mehr von der MRgFUS profitieren.
